# Uptake and Distribution of Cd in Sweet Maize Grown on Contaminated Soils: A Field-Scale Study

**DOI:** 10.1155/2013/959764

**Published:** 2013-11-21

**Authors:** Wending Xu, Guining Lu, Zhi Dang, Changjun Liao, Qiangpei Chen, Xiaoyun Yi

**Affiliations:** ^1^School of Environment and Energy, South China University of Technology, Guangzhou 510006, China; ^2^The Key Lab of Pollution Control and Ecosystem Restoration in Industry Clusters, Ministry of Education, Guangzhou 510006, China

## Abstract

Maize is an economic crop that is also a candidate for use in phytoremediation in low-to-moderately Cd-contaminated soils, because the plant can accumulate high concentration of Cd in parts that are nonedible to humans while accumulating only a low concentration of Cd in the fruit. Maize cultivars CT38 and HZ were planted in field soils contaminated with Cd and nitrilotriacetic acid (NTA) was used to enhance the phytoextractive effect of the maize. Different organs of the plant were analyzed to identify the Cd sinks in the maize. A distinction was made between leaf sheath tissue and leaf lamina tissue. Cd concentrations decreased in the tissues in the following order: sheath > root > lamina > stem > fruit. The addition of NTA increased the amount of Cd absorbed but left the relative distribution of the metal among the plant organs essentially unchanged. The Cd in the fruit of maize was below the Chinese government's permitted concentration in coarse cereals. Therefore, this study shows that it is possible to conduct maize phytoremediation of Cd-contaminated soil while, at the same time, harvesting a crop, for subsequent consumption.

## 1. Introduction

Heavy metal contamination of agricultural soils is a worldwide problem [1], and in China the problem is especially serious. If all of the low- or moderately-contaminated croplands were removed from agriculture production, then it would be impossible to meet the Chinese people's food needs. Cadmium (Cd) is one of the most mobile and bioavailable heavy metals in soil and can have eco-toxicological impacts on many organisms, including humans, plants, and animals, even at low concentrations [2, 3].

Maize is a familiar agricultural crop that is widely adapted to regions of China and can be cultivated easily. It has greater dry-mass than many heavy metal hyperaccumulative plants, such as Thlaspi caerulescens and Arabidopsis halleri. The roots and straws of maize can accumulate many kinds of heavy metals, including Cd, from contaminated soil. Fortunately, the seeds and fruits from maize generally accumulate metals at lower concentrations than leaves or roots [1]. After harvesting seeds and/or fruits from the plants, the straw, and roots, with their load of toxic metals, can be removed from the contaminated soil. With each successive growth cycle, the contaminated soil becomes cleaner than before.

One of the species used in the present study was sweet maize CT38, which was screened from many maize cultivars as an optimal cultivar for heavy metal by pot test [4]. The other cultivar was sweet maize HZ, which was the most wide cultivar due to its seed as delicious food for people and its straw as cow's feed. In order to verify the capability of accumulating Cd in field condition, we conducted this research for learning about the uptake and distribution of Cd in two sweet maize cultivars grown on Cd contaminated soils.

In order to enhance the efficiency of phytoextraction, amendments are widely used to increase the root uptake of metals through metal solubilization, and substantially increasing the speed of transfer of metals to shoots [5]. Chelating agents are the most commonly used amendments in chemically assisted phytoextraction of metals from soils [6–8]. However, the high metal-binding capacity with strong recalcitrance in biodegradation and their potential leaching to groundwater pose severe environmental concerns on the use of chelators, such as ethylenediaminetetraacetic acid (EDTA) [9, 10]. Therefore, the readily biodegradable [S,S]-ethylenediaminedisuccinic acid (EDDS) and nitrilotriacetic acid (NTA) are often used in enhanced phytoextraction investigations with fewer residual effects on the environment than EDTA [11, 12]. Zhou et al. [13] had found that cheap NTA had the similar capability for improving the heavy metal uptake by maize plant to costly EDDS without secondary pollution. So, in field scale, the chelator NTA has more feasibility for enhancing the maize remediation than EDDS. Several researchers [11, 14, 15] believed that the concentration and day of addition of chelants affected the accumulation of metals in plant tissues. Therefore, in order to improve the capability of Cd phytoextraction from the Cd-contaminated soils, the dosage of NTA and the time for adding them also need further study [16].

**Figure 1:**
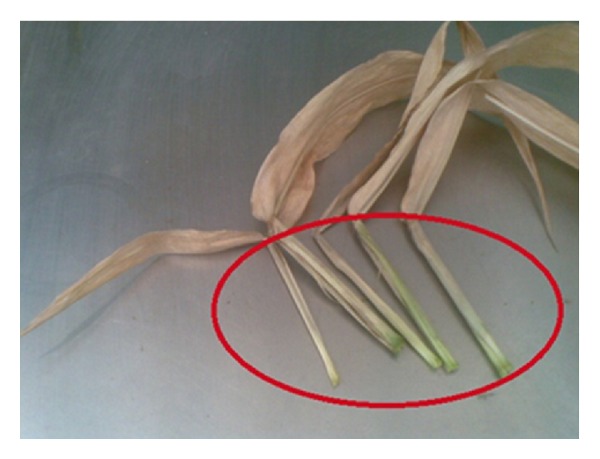
Sheaths (inside the ellipse) and laminas (outside the ellipse) of maize leaves.

Researchers have found that maize can take up Cd from contaminated soil and that different organs in the plant accumulate Cd at different concentrations [1, 17, 18]. For the maturing maize plant, leaves and roots accumulate the most Cd; the fruit accumulates the least. There are conflicting reports in the literature about whether leaves or roots accumulate the greater amount of Cd [17, 19–22]. We have undertaken research to clarify and enhance the uptake and distribution process of Cd by maize. Maize is composed of different organs, including root, stem, leaf, flower, and fruit. Every piece of leaf consists of three parts, namely, lamina, ligule, and sheath. Because the ligule has a very small mass compared to the masses of lamina and sheath, we have ignored it in our study. There are significant differences between the sheath that encases the stem and the lamina that extends from the stem (Figure 1). In contrast to many other studies [17, 21], we differentiate between the sheath and lamina portions of the leaf in order to get a more detailed picture of the distribution of the absorbed Cd.

The aim of this study was to investigate the uptake and distribution of Cd in sweet maize enhanced by NTA under the field condition. The objectives were (1) to compare the potential of Cd phytoextraction by two sweet maize in field, (2) to confirm the distribution of Cd in different tissues, and (3) to investigate the impact of NTA on Cd uptake and distribution.

## 2. Experimental Section

### 2.1. Experimental Site and Test Design

The experiment was conducted on a Cd-contaminated field in Hengli village, belonging to Renhe town, which is near the Guangzhou Baiyun airport in Guangdong, China. The region has a mild and warm climate (22.8°C mean temperature) with an annual average rainfall of 1982.7 mm. The soil type is a latosolic red soil with a loamy and silty texture typical of the region. The Cd contamination originated primarily from the disposal of city waste, including batteries, 30 years ago, a practice originally designed to increase the fertility of the reclaimed soil. While the fertility of the reclaimed soil was improved, Cd contamination was introduced into the soil at the same time. The concentrations of Cd in the ploughed topsoil were approximately 1.4 mg kg^−1^_._ Selected soil characteristics and heavy metal contents were shown in Table 1.

The test area consisted of eight 6 m × 8 m rectangular plot areas that were cultivated with two kinds of sweet maize from August 3 to October 25, 2007. The eight plots were divided in two groups: one group was planted with sweet maize CT38 and the other group was planted with sweet maize HZ. The test design and treatment codes are summarized in Table 2. The pollination time for both varieties of two maize is about 50 days from the sowing. Each group consists of four plots which had different amounts of NTA added (low and high) and/or different times of such addition (before and after pollination). The NTA treatments involved using appropriate volumes of a standard 200 mM NTA solution and injecting them in circular patterns around each plant at a distance of 10 cm from the plant stem.

### 2.2. Sampling and Sample Analysis

The sweet maize was harvested 75 days after the sowing date. Three maturing maize plants from each treatment group were chosen at random for analysis. Roots were excavated and washed to remove adhering soil. The shoots were immediately divided into stems, lamina, sheath, and fruits. The samples were packed into plastic bags and immediately transported to the laboratory where they were washed carefully with distilled water to remove any soil, cut into pieces, and then oven-dried for 1 hour at 105°C and for an additional 24 hours at 70°C. All dried materials were ground to 0.5 mm size using a centrifugal mill. Subsamples (1 g) were microwave-digested in 10 mL of HNO_3_ (65%) and 5 mL of H_2_O_2_ (30%). The digests were diluted to 50 mL with high-purity water and filtered. The filtrate was analyzed for Cd by inductively coupled plasma mass spectrometry (ICP-MS, Agilent 7500A) [1, 17]. The maximal relative standard deviation of triplicate measurements of the reference samples was 10%, and the respective maximal relative bias was 5% for Cd.

### 2.3. Statistical Data Analysis

The Cd concentrations from the different treatments were analyzed by ANOVA and a post hoc Bonferroni test, using Excel (version 2007), Origin (version 8.0), and SPSS (version 17) software. Data were assessed for accuracy and precision using a quality control system that included reagent blanks and triplicate samples. The precision and bias of the chemical analysis was less than 10%. Duncan's multiple range test at *P* ≤ 0.05 was used for mean separation.

## 3. Results and Discussion

### 3.1. The Dry Weight of Different Organs

All of the plants, irrespective of genotype or treatment, appeared to have normal growth. The dry-mass biomasses of the different organs in the plants are shown in Table 3. The stem was the heaviest in all organs, followed by the leaf, including both the lamina and sheath. The dry root was the lightest. The lamina was about two times heavier than the sheath. The two genotypes have the similar dry-mass characters, although the dry-mass of the CT38 plants was about 9% greater than that of the HZ plants. As shown in Table 4, the different organ mass ratios are similar for all of the treatment groups and both genotypes.

### 3.2. Total Cd Accumulation per Plant in Different Treatments

**Figure 2:**
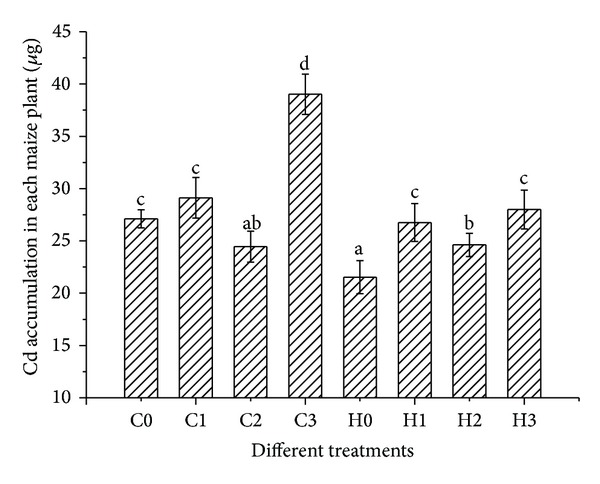
The total Cd accumulation per plant under different treatments. All values are mean ± SD (*n* = 3). Values labeled with different letters are significantly different from each other at *P* ≤ 0.05. The codes for different treatments in horizontal axis were correspondent to Table 2.

The overall evaluation of field scale phytoremediation depends on the total Cd accumulation per plant and total maize biomass in the field. The different maize treatments can result in differences in the uptake of Cd from the soil. The total Cd accumulation per plant under different treatments are shown in Figure 2.

According to Figure 2, CT38 was a better Cd accumulator than HZ; cf., C0–H0. This difference disappeared with prepollination treatment with NTA; cf., C1–H1 and C2–H2. With postpollination NTA treatment, the difference was significantly enhanced; cf, C3–H3. The postpollination application of NTA also had the greatest effect on the Cd accumulation by CT38 itself; cf. C0–C3. In contrast, the effect of NTA on HZ was its greatest when applied in the prepollination stage; cf., H0–H1 and H0–H2. 

Probably, pollination can make maize CT38 have more hormones which can adjust the combination and transportation of dry mass [23, 24]. Zn is the essential micronutrient element composing some enzyme, which is a special protein which belongs to the dry mass and can make important affections of biochemical reactions. Cd is a mimic of Zn and can transfer with the micronutrient by the way of water absorption. Therefore, we can draw a picture that after pollination, the momentum transferring dry mass from lamina and sheath to the ear and then to the fruits affirmatively causes the Cd shifting from lamina to sheath. However, before pollination especially before the appearance of ear, the micronutrient element and water mainly transfer from the root to the stem, to sheath, and then to the lamina. To the maize CT38, the chelator NTA improves the total Cd accumulation which mainly happens after pollination while maize HZ before pollination. The phenomenon means that for different maize genotypes, there are different fortifying measure even the same chemical chelator in order to get to the best accumulation affection.

### 3.3. The Cd Concentration in Maize Dry Fruits

As a widely planted coarse cereal crop, maize is important to mankind. The Cd concentration in seeds is of significant concern in food safety because the maize fruit is used for food and oil for human consumption and for forage for livestock.

**Figure 3:**
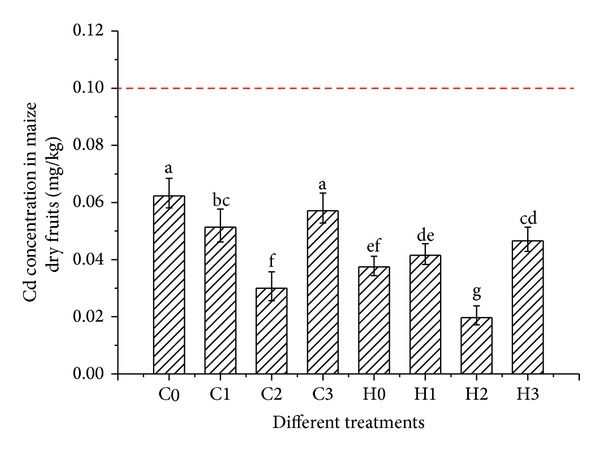
Cd levels in maize HZ dry seeds grown under with different NTA treatments. The broken line is the maximum permissible concentration of Cd in Chinese coarse cereals including the maize seeds (0.1 mg/kg, dry weight) (GB2762-2005) [25].

From Figure 3, we can find that the Cd concentration in edible part of seeds did not exceed the maximum permissible concentration of Cd in coarse cereals (0.1 mg/kg, dry weight), which was set by the Ministry of Health of China (GB2762-2005) [25]. The maize genotypes not only accumulate Cd in not edible maize straw including leave, sheath, stem, and root, but also the edible fruit part is safe enough because Cd concentration is under the permissible concentration of regulation. Other researchers also have found that the grain accumulates Cd less than other parts of maize plants [1, 17, 18]. 

In particular, even though CT38 maize with postpollination NTA treatment concentrates Cd significantly more than HZ maize, the CT38 grain remains at a level that is safe for human consumption. Furthermore, maize can be easily planted in a variety of agricultural situations, which leads to lower planting costs and greater economic benefit than other hyper accumulator plants such as Solanum nigrum L and Sedum alfredii. In China, arable land is limited, so that low and even some moderately contaminated lands remain in production because of the great need for food to feed 1.3 billion people. Therefore, the CT38 maize genotype with NTA postpollination application is a strong candidate for phytoextraction of Cd from contaminated soil since the land can continue production even while it is being cleansed.

### 3.4. Cd Distributed Fraction in Different Organs

**Figure 4:**
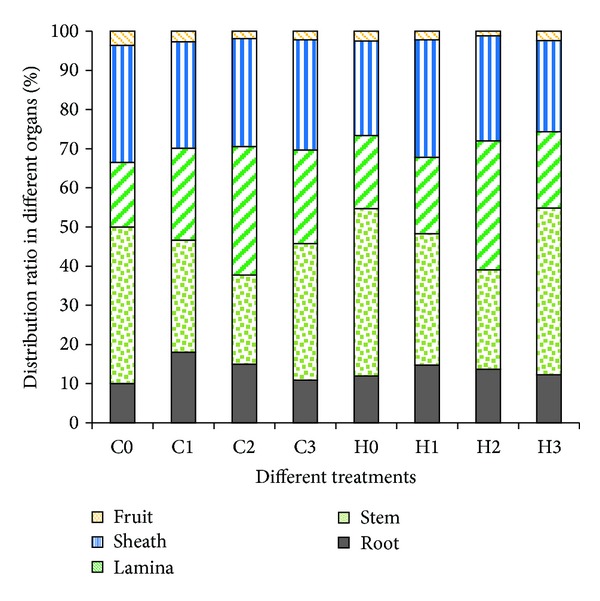
The Cd distribution fractions in different treatments. The leaf fraction is equal to the sum of the sheath and the lamina fractions.

The distribution of Cd in sweet maize can provide insight on how maize accumulates the metal (see Figure 4).

The percentage of Cd accumulated in the different organs of maize HZ is similar to that of maize CT38. For prepollination application, increasing amounts of NTA lead to increased relative Cd accumulation in the leaves (lamina plus sheath) (cf. C0 < C1 < C2 and H0 < H1 < H2) and decreased relative Cd accumulation in the stem (cf. C0 > C1 > C2 and H0 > H1 > H2). This behavior results from the prepollination growth emphasis on the nutrition organs such as the leaf, the main photosynthesis organ. The NTA chelator increases the Cd transport so that the metal can move with the water from the soil to the leaf, where it is deposited during transpiration. As the leaf biomass increases, the relative distribution of Cd in the leaf also increases.

Figure 2 shows that the total Cd accumulation with the lower NTA treatment was greater than that with the higher NTA treatment; that is, C1 > C2 and H1 > H2. The phenomenon indicates that there is an optimal amount of added chelator for enhancing the phytoremediation; this is not a case of “The more, the better.” 

Figure 4 shows that the ratio of Cd in the leaf to Cd in the stem (Cd_L_/Cd_S_) increases with the increasing amount of NTA added. The fact that the Cd_L_/Cd_S_ value after pollination is very close to the control treatment, probably means that the fraction of maize accumulating Cd may be related to the morphologic character. Before pollination, the stem is being in the protraction time, while after pollination the stem has already got to the highest and the plant producing point focuses on the ear and fruits. Even if the total accumulation quantities are significantly higher after pollination than control treatment (Figure 2), the fraction in different organs is very close between after pollination (C3 and H3) and control treatment (C0 and H0) for both of the two maize genotypes. Compared with the control treatment, adding same quantity of NTA after pollination treatment changes the percentage slightly more than before pollination. It is generally believed that after pollination, the maize grains are filled day after day but its nutrient organs (leaf, stem and root) have no significant growing change. The nutrient organs usually grow up under the regulation of plant hormones which have little quantities but very important in the course of the plant's growing up [26]. In different growth phases, maize has different planting center, where the plant hormones regulate the nutrient elements transfer and balance and then increase the dry matter faster. What on earth, the plant hormone regulates the distribution and transfer of Cd element? We need to do more study to find the truth in order to use the rule to clean up the Cd pollution in soil.

### 3.5. Cd Allocation in Different Organs of Two Maize Genotypes

The concentrations of accumulated Cd in different parts of the plant grown under different treatments are listed in Table 5. This information can help us understand how the maize accumulates the Cd through in the entire period of growth.

#### 3.5.1. The Contrast of Two Maize Genotypes without NTA Addition

The highest Cd concentration in both genotypes occurred in the sheath. The Cd concentration decreased in the following sequence: sheath > root > lamina > stem > fruit (Table 5). In the last line of Table 5, the results for sheath and lamina are combined into an overall amount for the leaf. In this case, there is a distinction between CT38 and HZ; that is, for CT38, leaf > root > stem > fruit; while for HZ, root *≈* leaf > stem > fruit, where the difference between root and leaves for HZ is not significant at the *P* < 0.05 level. The different sequences between root and leaf might be related to differences in the maize genotypes and/or different growing conditions, such as subtlely different soil. Whether or not the sequence sheath > root > lamina is the same for all mature Maize genotypes in field condition needs further study to clarify. 

#### 3.5.2. Maize CT38 Accumulation of Cd with NTA Addition before Pollination

The data in Table 5 show that the sheath is the highest Cd-enriched organ and fruit is the lowest, irrespective of the addition of NTA before pollination; that is, sheath > root > lamina > stem > fruit. With the NTA addition increasing, the Cd concentration in Maize's different organs have different changing trend. For the sheath and fruit, C0 *≈* C1 > C2; lamina, C2 *≈* C1 > C0; stem, C0 > C1 > C2; and root, C1 > C2 > C0. Lamina and sheath have the reverse tendency, though both of them belong to the overall leaf organ. We compare the Cd concentration between the root and the overall leaf (last line in Table 5) and find all probable occurrences to happen. No NTA adding (C0 treatment), the leaf is higher than root; adding in 25 mmol NTA each Maize plant before pollination (C1 treatment), the Cd concentration increases much more in root than in leaf and eventually the root is higher than leaf. However, in the condition of NTA adding in 100 mmol NTA each Maize plant before pollination (C2 treatment), almost all the organs of Maize CT38 accumulate Cd less than C1 treatment except lamina. Cadmium is transported from soil to plant roots by mass flow, diffusion, and interception. Mass flow and diffusion are considered to be the most important supply mechanism for ions in soil. Mass flow means that ions dissolved in soil solution are transported to the roots with the transpiration flux [3]. As a biograde chelator, NTA increased the solubility (NaNO_3_-extraction) of Cd ions by factor of 58 [27] and can increase shoot metal concentrations by a factor of 2-3 [28]. Root is the part under the ground and the soil solubility Cd can damage the root more easily. Before the pollination, the root is more sensitive to the NTA adding than the leaf. Too much NTA application (C1 treatment) may damage the Maize root directly and slow down the normal growing course, and then cause Cd concentration descending in all other organs.

#### 3.5.3. Maize CT38 Accumulate Cd in Different Growing Phases

In contrast with no NTA adding treatment, NTA adding in 25 mmol each maize before pollination makes the Cd concentration become higher in root and lamina, but not significantly changing in sheath. However, after pollination, NTA adding in 25 mmol each maize can make the sheath organ accumulate Cd in higher concentration. After pollination, the Maize is in reproductive growth period and mainly increases the fruit organ. Almost all nutrition is transported to the edible parts of the Maize; the Cd can also be transferred from soil to above ground organ following with the nutrition elements, such as Zn and Fe. Seeds and fruits generally accumulate metals at lower concentrations than leaves or roots [29, 30]. Some researchers [29] found Maize seed produced on contaminated land may be suitable for animal feed, and the other researchers [31–33] thought the stems and leaves could be used for nonfood purposes such as bioenergy production. Before pollination, the mass fluent is mainly from the root to stem, to sheath, terminally to lamina. However, after pollination, the mass fluent is focused on the fruit organ, namely, corn cob. According to the Maize's special structure, dry mass will transfer from the lamina to the sheath and then to the stem, then eventually transfer to the corn cob. Maybe the Cd accumulation is related to the dry mass accumulation. Whether the sheath and stem are accumulating more Cd after pollination than before pollination with the same NTA addition is a common rule for all Maize or not, it needs further test and theory study to be clarified. 

From Table 5, we can find that there are the contrary root leaf contrast effects between the C1 and C3. Before pollination root, is higher than leaf, however, after pollination, leaf is higher than root. Meanwhile, we can find the Cd concentration in leaf overrun that in the root is mainly depending on the distinct increasing in the sheath. For C1 and C3, even though there are different contrast effects between leaves and roots; however, we found that the sequences (sheath > root > lamina) are same. Maize leaves are divided into repeated longitudinal units consisting of vascular tissue, bundle sheath, and mesophyll cells [34]. Even though the sheath and lamina both belong to the part of the leaf, they have distinct structure and cell composition. In sheath, the main composition is the bundle sheath. But to the lamina, the mesophyll cell is the main composing ingredient. Sheath have more Cd concentration than lamina, which means that bundle sheath cells can deposit more Cd than mesophyll cells in the maturing Maize. So, we think that the capability of sheath and lamina is closely related to their chemical composition and molecular structure. 

#### 3.5.4. Maize HZ in Different Adding Concentration before Pollination

From Table 5, we find that the similar distribution sequences on the different organs also happened for Maize HZ: sheath > root > lamina > stem > grain. Same as Maize CT38, adding NTA can change the Cd concentration in different Maize HZ organs; however, the relatively sequences are not changed by NTA adding. We combine the lamina and sheath into the overall leaf and compare with other organs. For the treatment H1, low NTA addition for Maize HZ, Cd concentration in leaves is lower than roots but they have no significant difference (*P* < 0.05) while the treatment H2, high NTA addition for Maize HZ, Cd concentration in leaves is higher than roots and they also have significant differences (*P* < 0.05). For H1 and H2, there are opposite contrast effects between leaves and roots, however the sequences (sheath > root > lamina) are still the same. Sheath has the more Cd accumulation than lamina; even both of them belong to the overall leaf organ. From Tables 3 and 5, we can find that the low NTA adding is more reasonable than high NTA adding in order to make much Cd accumulating in the shoot. 

#### 3.5.5. Maize HZ Accumulate Cd in Different Growing Phase

From Table 5, we find that Maize HZ has different characters with Maize CT38. For Maize HZ, in contrast with adding NTA before pollination, adding NTA after pollination cannot increase the Cd concentration obviously in sheath. However, for Maize CT38, Cd concentration in sheath increases significantly after pollination. What is more, for Maize HZ, Cd concentration in leaf has the slightly descending, not significantly, but for Maize CT38; Cd concentration in leaf has the obviously ascending after pollination. Therefore, the affection coming from pollination is hardly related to the Maize genotypes. Though there are distinct differences between the two Maize genotypes' shoot, the two Maize genotypes have same tendency in root character. Before pollination, adding NTA with low concentration treatment (C1 and H1), the Cd concentration is the highest than the other treatments. And after pollination, adding NTA with low concentration treatment (C3 and H3), root accumulates Cd less than before pollination. As a high biomass crop, Maize has the well-developed root system in favour of Cd phytoextraction. The Cd^2+^ enters the shoot mainly through the root compared with the air absorb and dust settlement. Before pollination, in the root developing period, many essential elements enter the root from the soil solution; which are deleterious to all organisms, including plants [35, 36] and human beings [37], but zinc is the necessary element [38]. Unfortunately, Cd is a dangerous zinc mimic and can enter the plant following with Zn [2]. Pollination means Maize plants enter the product course and all energy and dry matter focus on the fruits or seeds. Adding NTA after pollination means the soil exchangeable Cd increasing in the course of fruit forming. Dry matter can transfer from other organs to fruits and make the fruits become abundant. From Table 5, we can speculate on that the appetency of Maize can hold the Cd beside the seeds. Dry matter transfers from lamina to sheath, and the Cd was flowing with nutrition matter like glucose. According to the Maize plant's special structure, when the nutritional dry mass transfers from sheath to stem, Cd is refused outside of the stem knar and resorted in the sheath.

## 4. Conclusions

Maize is a strong candidate crop for us in phytoremediation of low- and medium-grade Cd contaminated farmland in China. Compared with maize HZ, the maize CT38 had more significantly greater accumulating capability and more dry mass. The Cd concentration in the various parts decreased in the following order: sheath > root > lamina > stem > fruit, whether NTA was used for fortifying the effect of phytoremediation or not. Both maize genotypes produced fruit with Cd levels that were under the maximally permitted amount by the Chinese standards (GB2762-2005), so the two maize genotypes could be used to treat Cd contamination in contaminated farmland while still producing a consumable crop. 

## Figures and Tables

**Table 1:** Selected physical and chemical characteristics of soil in the field.

Parameter	Value (mean ± SD, *n* = 3)
OM (%)	24.37 ± 5.26
pH	6.25 ± 0.19
CEC (cmol·kg^−1^)	31.51 ± 2.83
Available N (mg·kg^−1^)	179.85 ± 38.7
Available P (mg·kg^−1^)	82.73 ± 18.15
Available K (mg·kg^−1^)	313.17 ± 63.40
Total Cd (mg·kg^−1^)	1.432 ± 0.276
Extractable Cd (mg·kg^−1^)	0.315 ± 0.038

**Table 2:** Test design and treatments code.

Maize genotypes	No NTA	NTA		
		Before pollination		After pollination
		25 mmol NTA/plant	100 mmol NTA/plant	25 mmol NTA/plant
CT38	C0	C1	C2	C3
HZ	H0	H1	H2	H3

**Table 3:** The biomass weight of different maize organs grown with different treatments (dry mass weight, g). Root dry weight is only collected in the plough layer soil, the stem weight includes the above ground stem and the underground stem. All values are mean ± SD (*n* = 3).

Organ	C0	C1	C2	C3	H0	H1	H2	H3
Root	8.31 ± 0.28	8.57 ± 0.20	8.34 ± 0.30	8.43 ± 0.13	7.62 ± 0.19	7.73 ± 0.17	7.58 ± 0.22	7.72 ± 0.09
Stem	78.09 ± 2.85	77.14 ± 3.41	77.85 ± 3.77	78.10 ± 1.54	71.94 ± 1.54	70.63 ± 0.58	71.80 ± 0.30	70.92 ± 2.09
Lamina	20.58 ± 2.53	20.42 ± 1.19	20.57 ± 1.72	20.14 ± 0.05	18.95 ± 1.21	19.08 ± 2.93	18.79 ± 2.70	18.47 ± 1.21
Sheath	10.28 ± 0.98	10.22 ± 1.15	10.29 ± 2.60	10.08 ± 0.24	9.64 ± 1.00	9.80 ± 1.17	9.41 ± 1.77	9.23 ± 0.72
Seeds	15.65 ± 0.97	15.40 ± 1.86	15.48 ± 1.71	15.08 ± 2.63	14.38 ± 1.52	14.16 ± 0.72	14.30 ± 1.56	14.20 ± 0.95

**Table 4:** The biomass ratio of different maize organs with different treatments.

Organ	C0	C1	C2	C3	H0	H1	H2	H3
Root	6.3%	6.5%	6.3%	6.4%	6.2%	6.4%	6.2%	6.4%
Stem	58.8%	58.6%	58.8%	59.2%	58.7%	58.2%	58.9%	58.8%
Lamina	15.5%	15.5%	15.5%	15.3%	15.5%	15.7%	15.4%	15.3%
Sheath	7.7%	7.8%	7.8%	7.6%	7.9%	8.1%	7.7%	7.7%
Seeds	11.8%	11.7%	11.7%	11.4%	11.7%	11.7%	11.7%	11.8%

**Table 5:** Cd concentrations in different parts of different treatments (mg/kg). All values are mean ± SD (*n* = 3).

Organ	C0	C1	C2	C3	H0	H1	H2	H3
Root	0.326 ± 0.015	0.612 ± 0.039	0.439 ± 0.016	0.505 ± 0.031	0.339 ± 0.013	0.509 ± 0.019	0.445 ± 0.016	0.444 ± 0.014
Stem	0.139 ± 0.012	0.108 ± 0.009	0.072 ± 0.007	0.173 ± 0.015	0.128 ± 0.012	0.127 ± 0.011	0.087 ± 0.008	0.168 ± 0.015
Fruit	0.062 ± 0.005	0.051 ± 0.006	0.030 ± 0.005	0.057 ± 0.006	0.038 ± 0.003	0.042 ± 0.004	0.020 ± 0.004	0.047 ± 0.004
Lamina	0.217 ± 0.035	0.335 ± 0.016	0.392 ± 0.036	0.463 ± 0.037	0.213 ± 0.022	0.275 ± 0.015	0.427 ± 0.046	0.294 ± 0.037
Sheath	0.791 ± 0.090	0.771 ± 0.075	0.662 ± 0.072	1.086 ± 0.151	0.534 ± 0.082	0.813 ± 0.087	0.704 ± 0.061	0.709 ± 0.072
Leaf*	0.410 ± 0.038	0.482 ± 0.029	0.478 ± 0.031	0.671 ± 0.029	0.321 ± 0.026	0.462 ± 0.074	0.521 ± 0.033	0.432 ± 0.037

*Cd concentration in leaf is calculated from the fomula as *C*_leaf_ = (*M*_lamina_ × *C*_lamina_ + *M*_sheath_ × *C*_sheath_)/(*M*_lamina_ + *M*_sheath_), *M*_lamina_ and *M*_sheath_ means the dry mass weight of lamina and sheath, *C*_lamina_ and *C*_sheath_ means the Cd concentration of lamina and sheath. The dry mass weight of leaf *M*_leaf_ = *M*_lamina_ + *M*_sheath_.
